# Case report: Spontaneous abortion of monoamniotic twins at the third trimester of pregnancy in *Camelus dromedarius*

**DOI:** 10.3389/fvets.2023.1273791

**Published:** 2023-12-04

**Authors:** Mohammad Shamim Hossein, Young-Bum Son, Yeon Ik Jeong, Mina Kang, Seejin Lee, Alex Tinson, Woo Suk Hwang

**Affiliations:** ^1^UAE Biotech Research Center, Abu Dhabi, United Arab Emirates; ^2^Department of OBS/Theriogenology, College of Veterinary Medicine, Chonnam National University, Gwangju, Republic of Korea; ^3^Hilli E.T. Cloning and Surgical Centre Presidential Camels and Camel Racing Affairs, Al-Ain, United Arab Emirates; ^4^NorthEastern Federal University, Yakutsk, Russia

**Keywords:** monoamniotic twins, abortion, camels, late-term, pregnancy loss

## Abstract

Monoamniotic twins develop when a blastocyst spontaneously splits its progenitor cells, and each group of progenitor cells independently grows to become an individual. It is the rarest type of twin pregnancy and usually has significant developmental or congenital abnormalities, a higher rate of abortion, perinatal morbidity, and mortality. There is no information regarding monoamniotic twins in livestock species. Here, we reported a spontaneous abortion of monoamniotic twins in a dromedary camel at 278 days of gestation. Gonadorelin acetate (100 μg) was injected intramuscularly to induce ovulation in the recipient. A 7 days-old embryo produced by somatic cell nuclear transfer was transferred transcervically to the recipient. Early pregnancy was confirmed by an elevated level of serum progesterone followed by ultrasonography at 22 and 44 days after embryo transfer. A single sac was observed on 22 days while twins were evident 44 days after embryo transfer. Pregnancy was periodically monitored by the tail-up phenomenon. A ruptured fetal sac was observed on the ground having two fetuses. On autopsy, full-grown fetuses were found. Their bodies were separated. There was no congenital anomaly or any malformation in the fetuses. According to the reported chronology in human twins, we hypothesized that the blastocyst splitted before 13 days as it was monoamniotic and not conjoined. If the embryo splits within 4 to 8 days, it develops two amniotic sacs, and splitting after 13 days develops conjoined fetuses. To the authors’ knowledge, this is the first reported case of monoamniotic twin abortion in dromedary camels. This report will increase awareness among practicing veterinarians and camel breeders about twin abortions.

## Introduction

A proper understanding of reproductive physiology is the key element for the breeding management of any species. Dromedary camels are prone to pregnancy losses at various stages of gestation periods. Early pregnancy loss could exceed 50% by day 80 of pregnancy (1, 2). However, the incidence of mid to late-term pregnancy loss is below 10%. Although causes and risk factors of pregnancy loss need to be explored thoroughly in camels, endometritis, luteal insufficiency, embryonic and environmental factors are considered the leading causes of early pregnancy loss (1). In contrast, systemic infections or injuries are the major causes of late-term abortion (3).

Camels are montocous animals, and twin births are extremely rare in nature (4). If twin ovulation occurs in the same estrous cycle, twinning is possible (5). However, twining could be developed by embryo splitting at the early stages of embryogenesis (6). Twins that develop by splitting an early embryo are known as monozygotic twins, and they are identical to each other. Depending on the timing of embryo splitting, several kinds of monozygotic twins could develop. Among them, monoamniotic twins, twins that share a common chorionic and amniotic cavity, are extremely rare. In humans, monoamniotic twins usually die before 24 weeks gestation (7) due to prematurity, cord entanglement, congenital anomalies, or twin-to-twin transfusion. In the present study, we reported a spontaneous abortion of a monoamniotic twin at 278 days of gestation in camels. This report will increase awareness among researchers, practicing veterinarians, and camel breeders about twin abortions.

## Case presentation

Ear skin biopsy was performed to collect tissues from an elite dromedary camel, and fibroblast cell line was established to clone the camel (8–10).

Oocyte donor and recipient camels were maintained in a large-scale cloning facility of the UAE Biotech Research Center, Abu Dhabi, United Arab Emirates (24°20′26.1″N 55°45′20.3″E) under the supervision of veterinarians. Camels were synchronized and superstimulated using our laboratory standard protocol (10). Briefly, oocyte donors and recipients were administered intramuscularly a single injection of 5,000 IU and 1,500 IU of pregnant mare serum gonadotropin (PMSG) (Ceva, Libourne, France) and 500 μg and 100 μg of cloprostenol (Jurox, Rutherford, Australia), respectively. Both the donors and recipients were injected with 100 μg gonadorelin acetate (Vetoquinol, Paris, France) on the 9th day after PMSG injection. Twenty two hours later, oocytes were collected transvaginally, from the donors using ultrasonography (10).

**Figure 1:**
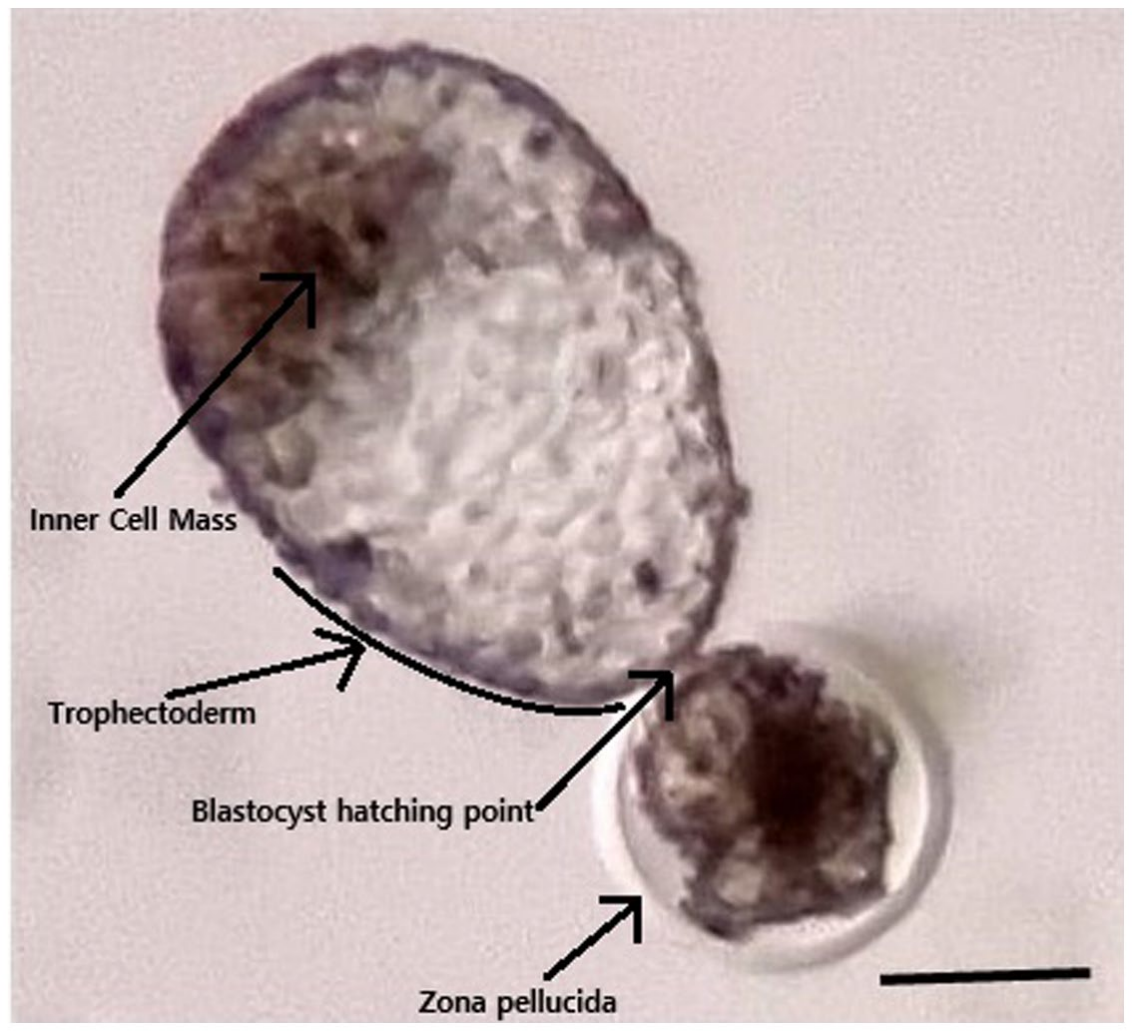
Photograph of the blastocyst transferred to the recipient. The blastocyst was 7 days old and started to hatch through the zona pellucida. The bar is 250 μm.

**Figure 2:**
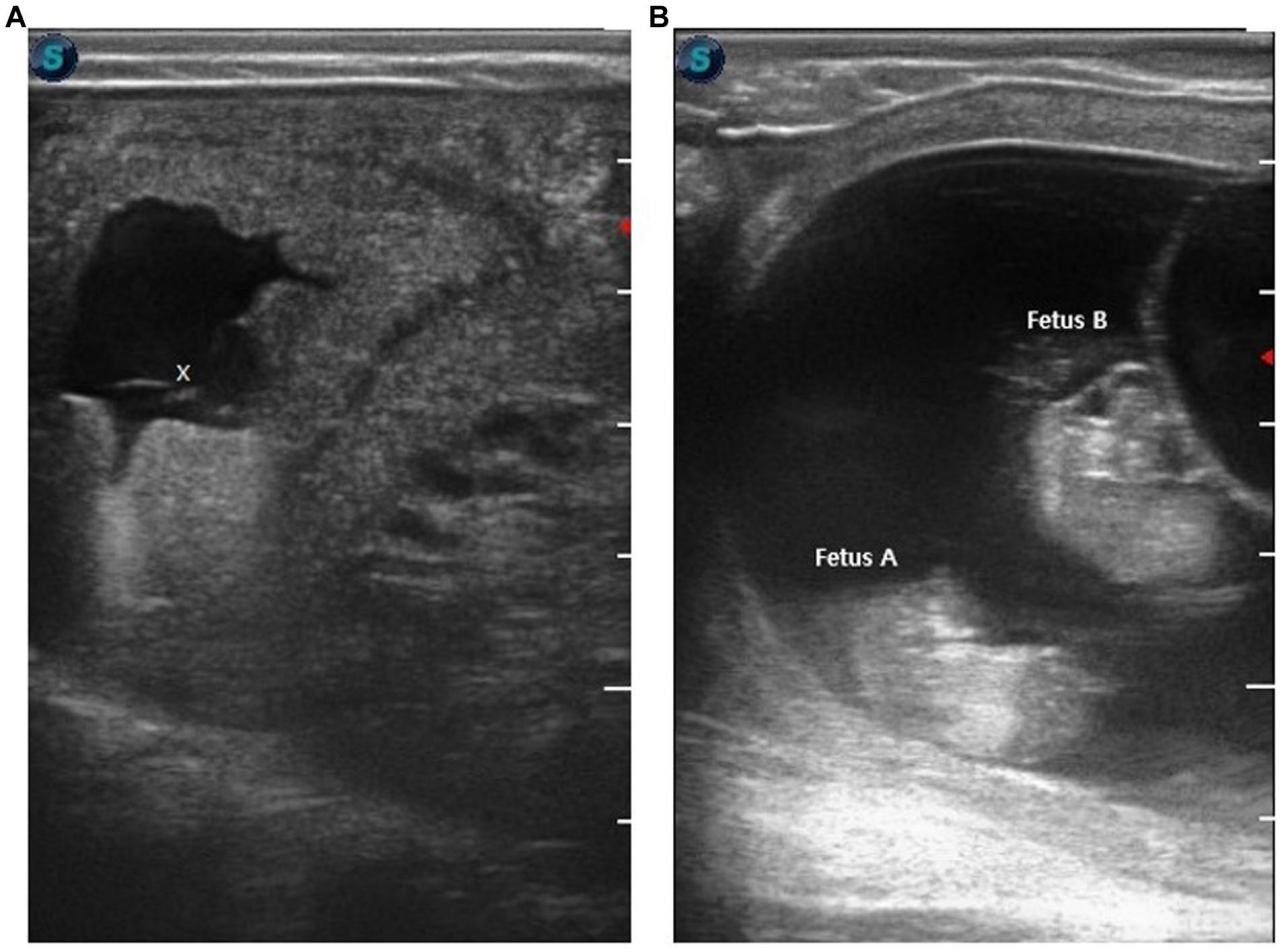
Ultrasonographic confirmation of pregnancy after transfer of cloned blastocyst. **(A)** The image shows a 17 mm cavity, 22 days after embryo transfer (x indicates fetus). **(B)** Ultrasonographic confirmation of twin pregnancy at 44 days after embryo transfer. The image shows a cavity having two fetuses; fetus A and fetus B.

Embryos were produced by injecting a diploid somatic cell into the enucleated metaphase II oocytes. The reconstructed oocytes were fused using electrical impulses in a fusion medium. After that, fused oocytes were chemically activated and cultured for 7 days for blastocyst production [for detailed procedure (10)]. The recipient (Multipara, body weight 470 kg) received a single 7 days-old embryo transcervically (Figure 1). Pregnancy was confirmed by measuring the serum progesterone level and by using ultrasonography 22 days after embryo transfer (Figure 2A). Serum progesterone level was 5.75 ng/mL and a single sac was observed on ultrasonography. On 44 days after embryo transfer, the serum progesterone level was 33.40 ng/mL and evidences of twining were revealed on ultrasonography (Figure 2B).

**Figure 3:**
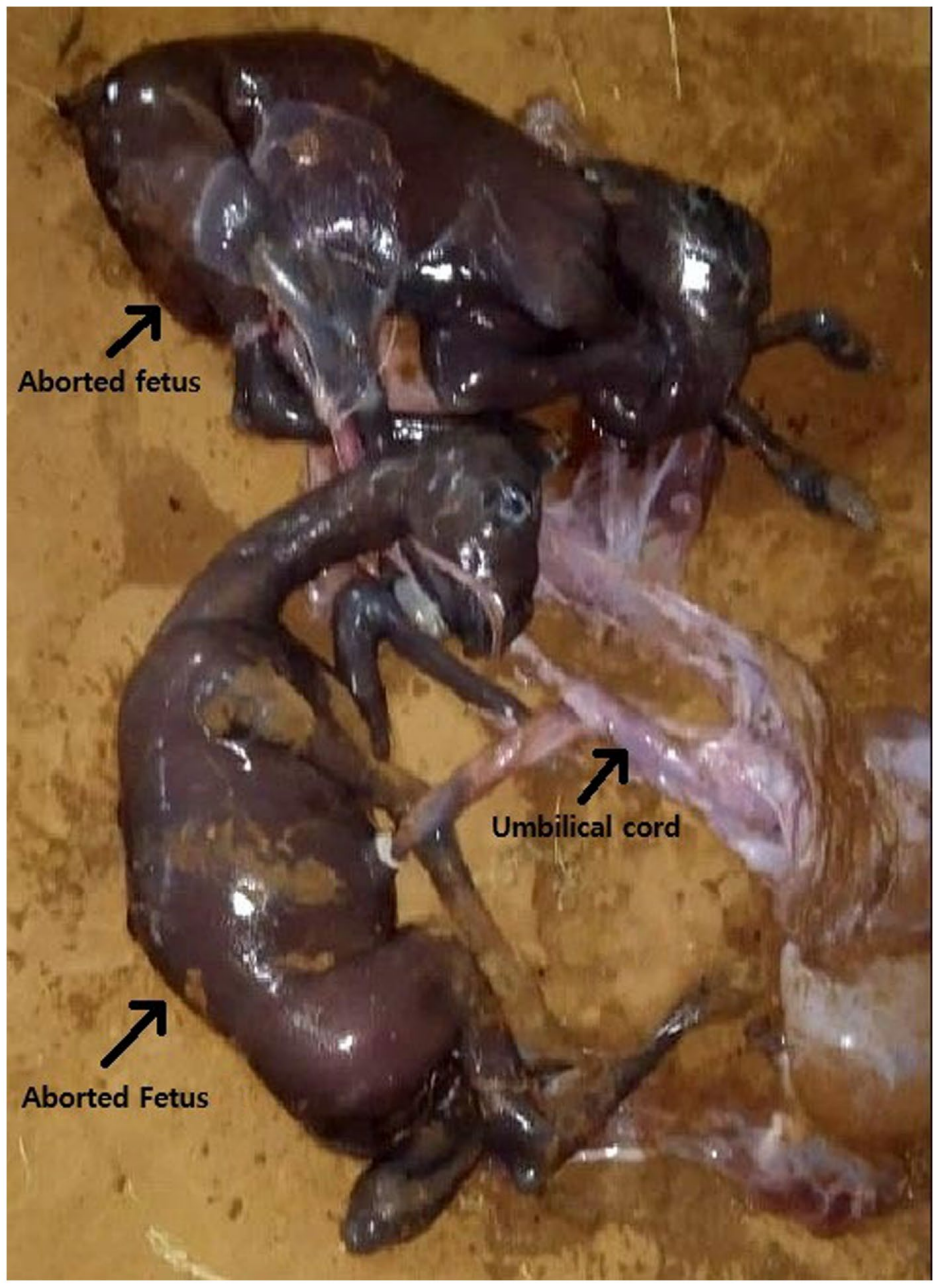
Aborted twin fetuses at 278 days of gestation.

The recipient spontaneously aborted twin fetuses on 278 days of pregnancy (Figure 3). The aborted fetuses were found on the ground in the morning. The recipient showed obvious signs of abortion. Hind legs were tinted with blood, and some portion of the placenta was hanging from the vagina. On autopsy, two full-grown fetuses were found. Their bodies weres separated. No congenital anomaly or malformation was seen in the fetuses. As the recipient aborted at late gestation, the brucellosis test was performed in fetal tissue and Dam’s blood, which was negative. Genomic DNA was extracted from aborted fetuses, recipient, and somatic cell donor using a Qiagen DNeasy Blood and Tissue Kit (Qiagen, Hilden, Germany) (8–10). Microsatellite analysis of 13 specific loci confirmed the fetuses as identical twins (Table 1).

**Table 1:** Microsatellite analysis of aborted twins, recipient, and cell donor.

Markers	Cell donor	Abortus 1	Abortus 2	Recipient
VOLP10	148/176	148/176	148/176	148/171
VOLP67	176/188	176/188	176/188	188/190
LCA63	238/240	238/240	238/240	240/240
LCA66	259/265	259/265	259/265	249/261
LCA90	149/165	149/165	149/165	137/145
CVRL01	225/225	225/225	225/225	225/229
CVRL05	144/167	144/167	144/167	144/144
CVRL07	212/220	212/220	212/220	214/222
LGU49	238/242	238/242	238/242	238/238
LGU75	105/107	105/107	105/107	105/113
YWLL44	218/224	218/224	218/224	202/234
P149	171/171	171/171	171/171	159/169
PCTD17	285/285	285/285	285/285	277/285

Microsatellite analysis was performed on genomic DNA from the aborted twins, recipient, and cell donor. The values of all markers were confirmed identical in aborted twins. Values represent the base pairs of the amplified microsatellite DNA markers in each sample.

## Discussion

Late-term pregnancy loss has severe economic consequences. Most of the pregnancy losses in camels occur in the first trimester of pregnancy; however, late-term pregnancy loss is also common (1). Here, we described a spontaneous abortion of twin fetuses at 278 days of gestation.

Camels have many fascinating reproductive phenomena, such as the induced ovulation by coitus, pregnancy only maintained in the left horn, higher rate of early pregnancy loss, especially in the case of twin pregnancies almost always one conceptus get reabsorbed within 60 days of pregnancy. Camels usually ovulate a single oocyte in each estrous cycle. However, around 14% of twin ovulation is reported; these twin ovulations normally result in twin pregnancies (4, 5). This kind of twin is known as a dizygotic twin, as two oocytes develop into two different embryos and is the most frequent form of twinning.

In the early zygotic stage, one embryo could be divided into two embryos and could develop into a monozygotic twin. In animals, there is no comprehensive study on the early development of monozygotic twins; however, different kinds of these twins were reported in humans, such as dichorionic-diamniotic twins, monochorinic-diamniotic twins, monoamniotic twins, or conjoined twins (11). The time of embryo division is the main determining factor for the type of twinning. In an elaborative study in humans, Hall (12) reported that most of the monozygotic twins develop within the first 7 days of embryonic life before blastocyst formation, and this early division gives a better survival chance to the twins. Monoamniotic twins develop when embryos divide after the blastocyst formation at around 8 to 13 days. Further delay in embryonic division exceeding 13 days develops conjoined twins in humans. We transferred a single 7 days-old blastocyst to the recipient, and we did not anticipate any twins. Therefore, the blastocyst divides after embryo transfer, and according to the reported chronology in human twins, this division seems to have occurred before 13 days as the twins were monoamniotic and not conjoined.

Artificial embryo splitting in the laboratory and the production of monozygotic twins have been reported in several livestock species, such as sheep (13), cattle (14), goats (15). Each of the splitted embryos implants separately, develops within its own sacs, and therefore increases fecundity. Spontaneous splitting of an embryo after blastocyst formation developed monochorionic and monoamniotic in nature. This kind of pregnancy has major discordant abnormalities, a higher rate of abortion, and perinatal mortality (7, 16).

The exact mechanism of spontaneous or natural embryo splitting and the development of monozygotic twins is not known. Illmensee (17) explains that the collapse of a blastocyst may results in the splitting of its progenitor cells and develop into a monozygotic twin. We assume that mechanical pressure on the blastocyst during its loading or transfer may be responsible for blastocyst splitting as we transferred a fully expanded hatching blastocyst. The findings of this case report will support the growing embryo transfer practices in camel breeding. A fully grown hatching or hatched blastocyst is usually transferred to the recipient in multiple ovulation embryo transfer and cloning programs (18). Proper care and gentle handling of blastocyst could prevent the development of monoamniotic twins.

In conclusion, twin or multiple pregnancies are always associated with high maternal complications such as abortion, preterm delivery, preterm pre-labor rupture of fetal sacs, etc., and fetal complications such as malformations, fetal growth restrictions etc. In humans, fetal reduction during multifetal pregnancy decreases the possibilities of spontaneous pregnancy losses and dramatically increases the survival benefit of the remaining fetus (19, 20). We recommend that if spontaneous resorption of fetal sacs does not happen in camels in the first trimester of pregnancy, selective sac reduction using lesions from human medicine technics could benefit twin pregnancy management in camels.

## Data availability statement

The original contributions presented in the study are included in the article/supplementary material, further inquiries can be directed to the corresponding author.

## Ethics statement

The animal study was approved by Management of Scientific Centers and Presidential Camels. The study was conducted in accordance with the local legislation and institutional requirements.Written informed consent was obtained from the participant/patient(s) for the publication of this case report.

## Author contributions

MH: Conceptualization, Writing – original draft. Y-BS: Conceptualization, Writing – original draft. YJ: Conceptualization, Writing – review & editing. MK: Conceptualization, Writing – review & editing. SL: Writing – review & editing. AT: Writing – review & editing, Conceptualization. WH: Conceptualization, Writing – review & editing, Supervision.
